# Growing of the TOR world

**DOI:** 10.1093/jxb/erac401

**Published:** 2022-11-15

**Authors:** Rossana Henriques, Maria Juliana Calderan-Rodrigues, José Luis Crespo, Elena Baena-González, Camila Caldana

**Affiliations:** School of Biological, Earth and Environmental Sciences, University College Cork, Cork, T23 TK30, Ireland; Environmental Research Institute, Lee Road, Cork, T23 XE10, Ireland; Max Planck Institute of Molecular Plant Physiology, Am Mühlenberg 1, 14476, Potsdam-Golm, Germany; Instituto de Bioquimica Vegetal y Fotosintesis, Consejo Superior de Investigaciones Cientificas (CSIC)-Universidad de Sevilla, Sevilla, Spain; Instituto Gulbenkian de Ciência, 2780-156 Oeiras, Portugal and GREEN-IT Bioresources for Sustainability, ITQB-NOVA, 2780-157 Oeiras, Portugal; Max Planck Institute of Molecular Plant Physiology, Am Mühlenberg 1, 14476, Potsdam-Golm, Germany

**Keywords:** TORC1, SnRK1, plant growth, phytohormones, metabolism, translation regulation, autophagy, development, nutrients, carbon partitioning, microalgae

**Twenty years have passed since the identification of the target of rapamycin (TOR) protein kinase in Arabidopsis. Research has expanded from functional characterization of the TOR complex (TORC1) and development of specific chemical inhibitors, to mapping the diverse biological relationships upstream and downstream of TORC1. New insights have been obtained on the mechanisms linking environmental perception to TORC1-mediated growth responses under optimal and stress conditions. Furthermore, molecular connections have been established between TORC1 and several phytohormone and nutrient signalling pathways (e.g. brassinosteroids, ethylene, and nitrogen), which together reinforce this pathway as a major hub controlling growth responses from unicellular algae to flowering plants.**

The TOR signalling pathway integrates nutrient, phytohormone, and environmental signals to adjust plant growth to the available resources. Its conservation among eukaryotes highlights its physiological relevance, which ranges from unicellular organisms to humans (González *et al.*, 2020; Liu and Sabatini, 2020). In this special issue we discuss some of the more relevant findings that were presented in the ‘Target of rapamycin (TOR) signaling in photosynthetic organisms’ EMBO workshop, which was held online in October 2021. The widespread use of high-throughput approaches including transcriptomics, proteomics, and metabolomics has widened our understanding of the evolutionary conservation, architecture, and physiological relevance of the TOR pathway within the green lineage. New upstream regulators, interactors, and downstream targets have been identified. In addition, the connection between environmental perception, nutrient status, and TOR activity was discussed at the workshop, as well as the emerging link between this pathway and the energy-generating machinery in the chloroplast. This exchange of ideas further cemented the notion that the TOR signalling pathway is crucial for survival of photosynthetic organisms (Fig. 1), and that although great progress has been made, there is still quite a long road ahead.

**Fig. 1.**
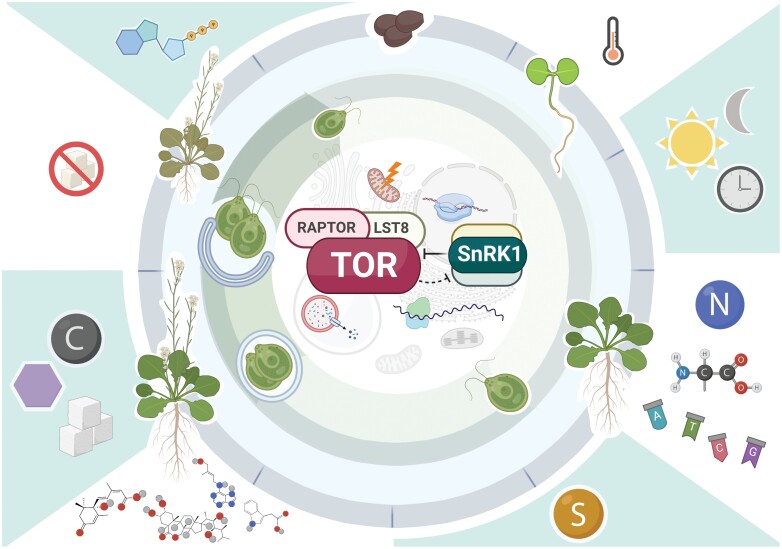
The green world of TOR: the main external and internal signals integrated by TORC1 and its antagonist SnRK1 complex regulate metabolic processes and developmental transitions in photosynthetic organisms. Starting at the top-left and moving clockwise around the outside of the figure: an ATP molecule; a thermometer representing temperature; the sun and moon representing light/dark cycles, and the circadian clock; nitrogen, amino acids, and nucleotides; sulfur; molecules of the hormones auxin, cytokinin, brassinosteroid, and abscisic acid; carbon, sugars representing sucrose, and a hexagon representing glucose; sugar starvation. These shaded and white spaces describe the biological processes integrated by the TORC1 signaling pathway. The outer circle shows the plant (Arabidopsis) developmental transitions controlled by TORC1/SnRK1. Starting at the top and moving clockwise: seeds; germinated seedlings; vegetative development; flowering transition; senescence. The next circle shows the algae (Chlamydomonas) cell cycle controlled by TORC1/SnRK1. Starting at the top and moving clockwise: a single cell; a growing cell; dividing cells; release of the daughter cells. The central part of the figure represents a cell and the metabolic processes regulated by TORC1/SnRK1. TORC1 is composed of TOR, Regulatory-Associated Protein of TOR 1 (RAPTOR), and Lethal with Sec13 protein 8 (LST8). The processes of respiration, transcription, translation, photosynthesis, and autophagy are highlighted inside the cell. Blocked lines indicate inhibition, and the dashed line implies a mutual inhibitory relationship that needs to be further characterized. Created with BioRender.com.

## TOR-complex architecture and signalling cascade in the green lineage

In animals, TOR assembles into two complexes with independent functions. TORC1 includes the master kinase TOR and its associated partners Lst8 and Raptor, whereas in TORC2, Raptor is replaced by Rictor/Avo3, and Sin1/Avo1 is present. However, photosynthetic organisms only possess TORC1, and it is likely that although TORC2 could have been present in the Last Eukaryotic Common Ancestor, it was lost early on during the evolution of the green lineage. This hypothesis is strengthened by the observation that Rictor homologs are present in some ancient microalgae (Mallén-Ponce *et al.*, 2022b). A detailed analysis of several microalgae genomes shows a high degree of conservation in TORC1 complex composition and the overall signalling cascade, including the canonical targets S6K1 and RPS6 (Mallén-Ponce *et al.*, 2022b). Interestingly, nutrient signal transducers found in animals such as small GTPases, which are missing in land plants, have been found in some glaucophytes and rhodophytes, suggesting the loss of specific TORC1 components at early stages of evolution. TORC1 is characterized as a promoter of anabolism (protein synthesis, ribosome biogenesis) and an inhibitor of catabolism (autophagy). These functions are conserved in the green lineage and the TOR-S6K1-RPS6 axis is involved in translation and polysome formation both in plants and microalgae (Couso *et al.*, 2020).

## Expanding the landscape of TOR upstream regulators

The main regulators of TORC1 activity have been identified and include auxin, light, nucleotides, and sugars (Ingargiola *et al.*, 2020; Artins and Caldana, 2022). However, in this issue we focus on recent findings on the molecular mechanisms underlying this regulation, both in microalgae and plants. TORC1 activity has been mostly assessed in response to specific treatments with either activator or inhibitor molecules and using S6K and RPS6 phosphorylation as read-outs of the pathway. This canonical approach has led to the identification of specific intracellular (e.g. carbon availability, amino acids, nitrogen, phosphorus) and environmental cues (light quality and duration) as regulators of TORC1, from microalgae to flowering plants (Liu *et al.*, 2021). In *Chlamydomonas reinhardtii* (hereafter Chlamydomonas), assimilated carbon, nitrogen, phosphorus, and acetate regulate TORC1 (Couso *et al.*, 2020; Upadhyaya *et al.*, 2020; Mallén-Ponce *et al.*, 2022a), whereas in Arabidopsis both far-red and blue light regulate TOR activity via COP1 to control cotyledon opening in de-etiolated seedlings (Chen *et al.*, 2018). Besides light quality, the duration of the light period also regulates plant growth since distinct photoperiods are associated with specific growth patterns in different organs. Defects in TORC1 components, such as in the case of *raptor1b* mutants, affect these patterns, especially in the roots. This interplay between day-length perception and TORC1 activity also relies on the functioning of the circadian clock (Henriques *et al.*, 2018; Urrea-Castellanos *et al.*, 2022). Carbon is assimilated in the Calvin–Benson–Bassham (CBB) cycle during photosynthesis and this in turn reflects on TORC1 activity in microalgae and plants. However, the main outstanding question remains: how is carbon availability perceived? Several reports have directly or indirectly addressed this question and there is a growing consensus that the abundance of specific amino acids (Ala, Leu, Val, Gln, and Glu) could increase TORC1 activity in microalgae as well as in Arabidopsis (Mallén-Ponce *et al.*, 2022b). In fact, inhibition of Leu biosynthesis in Arabidopsis results in higher levels of total branched-chain amino acids, such as Val, leading to TOR activation, increased numbers of meristematic cells of smaller size, and chloroplast developmental defects (Cao *et al.*, 2019; Schaufelberger *et al.*, 2019). In addition, nitrogen-starved Arabidopsis seedlings have higher TORC1 activity after treatment with different amino acids, with those involved in the TCA cycle and glycolysis showing the highest activation (Liu *et al.*, 2021). The increase in TORC1 activity by branched-chain amino acids also leads to higher actin bundling; however, this phenotype is maintained even in *raptor1b* mutants, suggesting that the TORC1 assembly is not required (Burkart and Brandizzi, 2021). Actin organization depends on TORC2, which is absent in plants. Therefore, these findings suggest that upon specific treatments, TOR might regulate the actin cytoskeleton in a plant-specific manner.

## The metabolic connection

Within the green lineage there are various strategies by which assimilated carbon is used for growth. In microalgae, carbon partitioning occurs within different cellular compartments, and storage takes place either in the form of starch or lipids (Mallén-Ponce *et al.*, 2022b). However, in plants there is a division between autotrophic/source organs (e.g. fully expanded leaves) that assimilate carbon, and heterotrophic/sink organs (e.g. roots, young leaves) whose growth relies on carbon supplied by source leaves that is exported as sucrose (Ingargiola *et al.*, 2020; Artins and Caldana, 2022). Moreover, these carbon-partitioning strategies also have to account for environmental cues, such as photoperiod, so that there is sufficiently available photosynthate for immediate growth while also ensuring that some will be stored as starch to sustain growth during the dark period. Coordination of starch synthesis and degradation rates is also under the control of the circadian clock. Recent findings have shown that TORC1 inhibition or sugar depletion can affect the circadian period, suggesting a tight correlation between this pathway and photoperiod perception (Zhang *et al.*, 2019; Wang *et al.*, 2020; Urrea-Castellanos *et al.*, 2022). Down-regulation of *TOR* transcript levels or lack of TORC1 components results in higher starch levels and an increase in triacylglycerides in Arabidopsis, as well as in Chlamydomonas and *Cyanidioschyzon merolae*, although an increase in amino acid metabolism and autophagy also occur in these microalgae (Jüppner *et al.*, 2018; Salem *et al.*, 2018; Pancha *et al.*, 2019). Pancha *et al.* (2019) also provided a mechanistic understanding on the connection between TORC1 and carbon reserves, showing that *C. merolae* TORC1 controls the phosphorylation of GLG1, a glycogenin involved in starch/glycogen synthesis. Further insights into TORC1 and carbon metabolism have come from the finding that the starch-deficient Chlamydomonas mutant *sta6* displays increased TORC1 activity (Mallén-Ponce *et al.*, 2022a).

## TOR and photosynthesis: mutual regulation?

The relevance of carbon assimilation via the CBB cycle and TORC1 activity has been well established by multiple studies showing a positive correlation between the two processes (Crespo *et al.*, 2022). On the one hand, down-regulation of photosynthesis reduces TORC1 activity in Chlamydomonas and *C. merolae*; on the other hand, inhibition of TORC1 signalling in microalgae affects the photosynthetic machinery (PSI and PSII) and components of the CBB cycle (Ford *et al.*, 2019; Couso *et al.*, 2021). Moreover, TORC1 modulates chloroplast function in *C. merolae* by affecting the transcription of nuclear and mitochondrial rRNA (Imamura *et al.*, 2018). In Arabidopsis, TORC1 induces the expression of chloroplast and photosynthetic genes, and its inhibition results in fewer and smaller chloroplasts. Moreover, modulation of TORC1 activity in Arabidopsis and rice also influences chloroplast development (Burkart and Brandizzi, 2021).

## Mining for TORC1 downstream targets: the power of combinatorial omics approaches

Recent advances in our understanding of TORC1 function in plants have been made possible by high-throughput analyses such as transcriptomics, proteomics, and phosphoproteomics (Pancha *et al.*, 2019; Van Leene *et al.*, 2019; Werth *et al.*, 2019; Scarpin *et al.*, 2020, 2022; Jamsheer K *et al.*, 2022a). These approaches have identified well-known interactors and targets, such as Raptor, S6K, and RPS6, as well as other expected regulators such as components of the translational machinery. In Arabidopsis, a comparative analysis of proteome and phosphoproteome data obtained by distinct TORC1 chemical inhibition highlighted the high specificity of each inhibitor treatment, with limited overlap being identified in targets or interactors (Jamsheer K *et al.*, 2022a). Interestingly, previously described interactions with transcriptional regulators (e.g. BZR1, EIN2, RBR1) were re-confirmed, but new connections with histone modifiers (e.g. histone acetylases and deacetylases) suggested that TORC1 could also be involved in the regulation of chromatin remodelling. In fact, upon glucose treatment, TORC1 is involved in heat stress protection and long-term recovery that depends on the accumulation of H3K4me3 positive marks in the promoters of thermomemory genes (e.g. *HSP21*). This process depends on Arabidopsis Trithorax 1 (ATX1), a H3K4 methyltransferase that is regulated by E2Fa, a TOR target (Meng *et al.*, 2022; Sharma *et al.*, 2022). These findings describe a role for TORC1 in modulating the transcriptional landscape, even if not directly.

The canonical role of TORC1 in regulating ribosome biogenesis and translation has mostly been associated with phosphorylation of S6K and RPS6. However, recent findings have shed light on the connection between TORC1 and ‘5´-terminal oligopyrimidines’ (5´TOP) mRNAs in Arabidopsis (Scarpin *et al.*, 2020, 2022). Although ribosomal proteins in animals possess a 5´TOP tract in their mRNAs and mTORC1 could be associated with translation of eukaryotic initiation factors such as eIF4B and eIF4G, in plants this regulation is not clear. However, the recent identification of Arabidopsis LARP1, a component of the initiation complex, as a TORC1 target involved in the translation of 5´TOP mRNAs has further strengthened the notion of high conservation of this signalling pathway between plants and animals (Scarpin *et al.*, 2020, 2022; Jamsheer K *et al.*, 2022a). Moreover, the targets of the conserved TORC1-LARP1-5´TOP module include mRNAs involved in ribosome biogenesis, mRNA translation, mRNA splicing, cell cycle progression, and vesicle trafficking. In addition, plant-specific 5´TOP mRNAs would include components of cell wall synthesis, plastid biogenesis, and developmental patterning. Complementing this analysis with the identification of novel 5´TOP mRNAs using data from previous reports (Nielsen *et al.*, 2019) has uncovered several candidates, such as cytosolic ribosomal proteins (e.g. eS6, eS7, uS11), elongation factors (e.g. eEF1Bγ1), and metabolic regulators (e.g. GRF3). Interestingly, strong 5´TOP mRNAs candidates also include FLZ proteins (e.g. FLZ3, 4, 5, 6, and 27) and bZIP transcription factors (e.g. bZIP1, 2, 11, and 44). Whereas the former are negative regulators of SnRK1 signalling, the latter act downstream of SnRK1 to promote stress reprogramming of transcription (Scarpin *et al.*, 2022). These findings shed light on the TORC1/SnRK1 connection, which is also discussed in this issue (see below).

Besides its critical function in translation, TORC1 could also modulate protein folding by interacting with HSP70, HSP60, and CPN60A. The latter could bind to Raptor 1B and regulate Rubisco folding/assembly (Jamsheer K *et al.*, 2022a). This analysis also confirmed other well-described targets such as YAK1 (Barrada *et al.*, 2019; Forzani *et al.*, 2019), and novel candidates for the TORC1 interactome would include PP2A and several enzymes involved in primary and secondary metabolism. Another group of interactors act as regulators of autophagy, such as the autophagy-related (ATG) proteins ATG1, ATG3, ATG1b, and ATG101. Other candidates identified in the TORC1 interactome and phosphoproteome analyses include the Arabidopsis Meiosis protein-2 Like (AML) RNA-binding proteins AML1, AML2, AML4, and AML5, RNA-splicing proteins, and components of the redox signalling pathway and of the ubiquitin-proteasome system (Jamsheer K *et al.*, 2022a). Taken together, these combinatorial strategies and analyses have contributed to expanding the ‘TORC1-tome’ and the challenge now is to functionally validate many of these candidates as *bona fide* TOR targets (Box 1).

## TORC1: a major signalling hub connecting different pathways

TORC1 is involved in different physiological responses as a result of crosstalk with other signalling pathways. Recent reports have connected TORC1 with the regulation of light-controlled RNA alternative splicing in conjunction with plastid retrograde signalling in the roots (Riegler *et al.*, 2021). Light and the circadian clock also seem to regulate RPS6 phosphorylation and this could be associated with a latitude-dependent photoperiod prediction mechanism (Enganti *et al.*, 2018; Panchy *et al.*, 2020; Urrea-Castellanos *et al.*, 2022). As noted above, TORC1 activity can also impact on circadian clock function but this seems to be a mutual connection, since core clock components could also modulate the TORC1 pathway (Li *et al.*, 2019; Wang *et al.*, 2020; Urrea-Castellanos *et al.*, 2022).

In addition, great progress has been made in dissecting the connections between TORC1 and several phytohormone signalling pathways (Meng *et al.*, 2022). Growth-promoting phytohormones (e.g. auxin, brassinosteroids, and gibberellins) are generally associated with high TORC1 activity, but the opposite is true for growth-restrictive hormones, for which a mutually inhibitory relationship with TORC1 has been described (e.g. ABA, ethylene, and JA). One such example is the TOR-mediated integration of glucose and ethylene signals into the control of hypocotyl elongation and root meristem activation. This process is mediated by phosphorylation of EIN2 by the glucose-TOR pathway at a site different from the one controlling the ethylene-CTR1-EIN2 axis (Fu *et al.*, 2021; Meng *et al.*, 2022). Furthermore, TORC1 and ABA regulate plant growth in opposite manners by exerting reciprocal regulation on each other (Gutierrez-Beltran and Crespo, 2022; Meng *et al.*, 2022). ABA represses TOR via SnRK2 and SnRK1 kinases whilst TOR represses ABA signalling via the PYL receptors, preventing them from triggering ABA signalling in the absence of stress (Wang *et al.*, 2018; Belda-Palazón *et al.*, 2020, 2022; Gutierrez-Beltran and Crespo, 2022; Meng *et al.*, 2022).

SnRK1 accumulation in different cellular compartments seems to be associated with specific functions (Gutierrez-Beltran and Crespo, 2022). For instance, it is sequestered by SnRK2s and PP2C phosphatases in nuclear repressor complexes. When these complexes dissociate in response to ABA, SnRK1 translocates to the cytoplasm to inhibit TOR activity (Belda-Palazón *et al.*, 2020, 2022). In addition, it has been proposed that the endoplasmic reticulum could act as an assembly platform for TORC1/SnRK1 mutual regulation and this would be modulated by FLZ proteins (Jamsheer K *et al.*, 2022b). Interestingly, as noted above, FLZs have also been identified as novel 5´TOP mRNAs under TORC1-LARP1 regulation, suggesting that they could play a dynamic role in TORC1/SnRK1 signalling. SnRK1 also associates with stress granules in the cytoplasm where it could mediate heat-stress responses (Gutierrez-Beltran *et al.*, 2021). Another process where TORC1 and SnRK1 play an opposite role is in autophagy (Liao and Bassham, 2020). Supporting this, several SnRK1-regulated ATG proteins (e.g. ATG1 and ATG3) have also been identified in the TORC1 interactome and phosphoproteome (Jamsheer K *et al.*, 2022a; Gutierrez-Beltran and Crespo, 2022), suggesting that TORC1/SnRK1 regulation could be present at the early stages of this process.

Besides its stress-related functions, SnRK1 plays a role in the daily maintenance of homeostasis, as well as in the coordination of metabolism and development (Peixoto and Baena-González, 2022). SnRK1 regulates central metabolism by direct enzyme phosphorylation and transcriptional control in what appears to be a core function in all organs and developmental stages: the maintenance of sucrose at optimal levels for growth (Peixoto and Baena-González, 2022). Tre6P (trehalose 6-phosphate) is considered to be a signalling molecule for sucrose availability that inhibits SnRK1 allosterically. A recent study has indicated that SnRK1 also contributes to Tre6P metabolism, linking sucrose and Tre6P in a manner that is not yet understood (Peixoto *et al.*, 2021). In addition, manipulation of SnRK1 impacts nitrogen metabolism, causing for example altered accumulation of amino acids and total protein. In seeds, SnRK1 promotes mobilization of lipid reserves during germination and seedling establishment (Henninger *et al.*, 2022).

Given the intricate connection between SnRK1 and TORC1, it is likely that many of the described outcomes are the result of a tug-of-war between these two regulators, where the perception of overall resources will ultimately determine which pathway is activated or inhibited.

Taken together the findings presented in this special issue strengthened the role of TORC1 as a signalling hub where multiple signals (e.g. nutrient and phytohormone levels, time of the day and season, light quality) are integrated to ensure temporal and spatial adjustment of plant growth and development by regulating a diverse range of specific effectors and targets.

**BOX 1. Future challenges**

- Although great progress has been made in uncovering the TORC1 interactome it will be important to functionally characterize many of the identified candidates that could function as alternative read-outs of the activity of this complex.
- Expanding our knowledge of TORC1 within the green lineage could uncover novel plant-specific functions commonly associated with TORC2 in mammals, and thus improve our understanding of growth regulation in model plants and crops.
- Although the glucose–TORC1 module has been extensively characterized in seedlings grown *in vitro*, it will be important to investigate the activity of this complex under standard plant-growth conditions (e.g. in the soil).
- Several findings point to a close connection between TORC1 and chloroplast function, development, and overall architecture; further dissection of this relationship could uncover how energy generation and consumption are balanced at the cellular level.
